# Integrating a gait analysis test in hospital rehabilitation: A service design approach

**DOI:** 10.1371/journal.pone.0224409

**Published:** 2019-10-30

**Authors:** Javier Marín, Teresa Blanco, José J. Marín, Alejandro Moreno, Elena Martitegui, Juan C. Aragüés

**Affiliations:** 1 IDERGO (Research and Development in Ergonomics) Research Group, I3A (Aragon Institute of Engineering Research), University of Zaragoza, Zaragoza, Spain; 2 Department of Design and Manufacturing Engineering, University of Zaragoza, Zaragoza, Spain; 3 HOWLab (Human Openware Research Lab) Research Group, I3A, University of Zaragoza, Zaragoza, Spain; 4 GeoSpatiumLab, S.L. Zaragoza, Spain; 5 Department of Health and Sports Sciences, University of Zaragoza, Zaragoza, Spain; 6 Rehabilitation and Physical Medicine Service, HUMS (Miguel Servet University Hospital), Zaragoza, Spain; University of Birmingham, UNITED KINGDOM

## Abstract

**Background:**

Gait analysis with motion capture (MoCap) during rehabilitation can provide objective information to facilitate treatment decision making. However, designing a test to be integrated into healthcare services requires considering multiple design factors. The difficulty of integrating a ‘micro-service’ (gait test) within a ‘macro-service’ (healthcare service) has received little attention in the gait analysis literature. It is a challenge that goes beyond the gait analysis case study because service design methods commonly focus on the entire service design (macro-level).

**Objective:**

This study aims to extract design considerations and generate guidelines to integrate MoCap technology for gait analysis in the hospital rehabilitation setting. Specifically, the aim is to design a gait test to assess the response of the applied treatments through pre- and post-measurement sessions.

**Methods:**

We focused on patients with spasticity who received botulinum toxin treatment. A qualitative research design was used to investigate the integration of a gait analysis system based on inertial measurement units in a rehabilitation service at a reference hospital. The methodological approach was based on contrasted methodologies from the service design field, which materialise through observation techniques (during system use), semi-structured interviews, and workshops with healthcare professionals (13 patients, 10 ‘proxies’, and 6 doctors).

**Results:**

The analysis resulted in six themes: (1) patients’ understanding, (2) guiding the gait tests, (3) which professionals guide the gait tests, (4) gait test reports, (5) requesting gait tests (doctors and test guide communication), and the (6) conceptual design of the service with the gait test.

**Conclusions:**

The extracted design considerations and guidelines increase the applicability and usefulness of the gait analysis technology, improving the link between technologists and healthcare professionals. The proposed methodological approach can also be useful for service design teams that deal with the integration of one service into another.

## Background

Although we are not conscious of its complexity, gait is a complex activity for human beings. It requires high motor control, and its pathologies have a harmful effect on personal autonomy and daily life activities [1]. Gait analysis with motion capture (MoCap) technology in the usual clinical practice is called ‘clinical gait analysis’ [2]; it is considered an important measurement in the rehabilitation field, where decision making on numerous treatments and interventions can benefit from objective information on the patient’s walking pattern [3–7]. Clinical gait analysis is especially important in the treatment of hemiparetic individuals after a stroke (or other causes), because they experience numerous impairments in walking skills that are reflected in the gait pattern [8].

In this regard, clinical gait analysis based on MoCap technology can be defined as the instrumented measurement of movement patterns that comprise walking and the associated interpretation of these patterns[2]. A MoCap instrumentation that is extensively used in biomedical research is optical technology (gold standard), which tracks the position of reflective surface markers with infrared cameras. Another instrumentation is technology based on inertial measurement units (IMU), which are electronic devices that measure rotations (rotation matrices, Euler angles, quaternions, etc.) by processing the signal of embedded sensors (accelerometers, gyroscopes, and magnetometers) [9–13]. Regarding their differences, optical technology is more accurate than IMU technology, which could present drift errors. However, it requires a camera infrastructure and sometimes presents shadowing problems [14]. In contrast, the IMU technology is more economical and portable and has been recently used in wearable technology [15], which could encourage cloud data processing and information exchange in the context of the Internet of things [16].

MoCap gait analysis is widely used in clinical research; however, certain factors have prevented the spread of this technology in hospital rehabilitation [17,18]. Nowadays, gait evaluation relies mostly on observational criteria. These systems involve high technical complexity, requiring the application of a strict protocol for accurate and repeatable measurements [2,19,20]. The massive amount of information they provide requires complex processing methods [8,21,22]. Currently, the professionals involved in this type of analysis must be highly qualified [2], which conflicts with the present needs for simplicity, usability, and intuitiveness [23].

Many of these factors have been identified by the cited researchers; however, despite being relevant, the difficulty of the ‘servitisation’ of gait analysis has received little attention in the literature [15]. Because of the challenges that servitisation poses, it should be added as an additional research objective.

Designing an integrated test in the biomedical field is particularly complex because multiple stakeholders have different needs. Solutions that involve all users (patients, doctors, therapists, etc.) in the context of the technology are required to make it useful, cost-effective, and truly usable [24]. This includes family members and people close to patients (proxies), who are especially affected by the situation that the patient experiences [25,26]. Therefore, we address a problem that goes beyond the development of the technology by investigating how to apply a certain technology (gait analysis) to a specific context (hospital rehabilitation services).

If we assume that the design object is a service and not only the technology that measures movement, the use of specific service design techniques [27,28] will allow us to predict failures, extract critical points, and consider the intangible and contextual part of the product [15]. These methods are aligned with the patient-centred care (PCC) philosophy, which is a priority in the healthcare field [29] and shares its philosophy with human-centred design (HCD) [30]. Despite the uncertainty in health services (timing, diagnoses, resources per patient, etc.), these methods are relevant for professionals to know exactly what their roles are and to work in a more optimal and orderly manner. To achieve this, the involvement of users in the design process is key [31].

However, these methods, although highly flexible, are commonly focused on the entire service design [27,30]. Service design methods and the theoretical basis of service-dominant logic [32] aim to guide service innovations and are focused on the perspective of the whole company. Wetter et al. [33] identified this situation as a ‘macro-level’ approach and asserted that, in some cases, it is not clear how to act at a more operational level with a ‘micro-level’ approach or how to guide specific actions and projects to favour service innovations. Thus, contributions are needed to connect design methods with real situations and problems, producing pragmatic, empirical, and micro-level approaches.

Based on this terminology, we face a scenario in which we incorporate what we call a ‘micro-service’ (a gait analysis test) within a more complex ‘macro-service’ (rehabilitation services). In terms of service design, this situation has particularities and implications that are essential to consider. How can the gait test be included in the hospital as an additional medical test? What additional materials must be designed, developed, or adapted for the gait test? How do the patient’s capabilities influence the design and guidance of the gait test? Which professionals will guide it? Is there a lack of certain professionals in the macro-service? In this regard, qualitative studies can provide a meaningful view of the perspectives, opinions, and priorities of the users (patients, healthcare professionals, and proxies) and reveal the underlying conceptual structure of their existing and/or desired interactions [34–36].

This article qualitatively evaluates a MoCap gait analysis system during its integration in a hospital rehabilitation environment, constructing the research through design methods. We focus on a specific case study of neurological patients with spasticity in the lower limbs who are treated with botulinum toxin. Thus, we present design considerations and guidelines for the improvement and adaptation of the system, which can be extrapolated to other scenarios at both the service design and hospital environment levels. In the results section, the guidelines are structured into six main themes. Finally, in the discussion section, we discuss the advantages and benefits that an integrated gait analysis test would introduce in rehabilitation services.

## Methods

A qualitative research design was used to provide design considerations and guidelines to integrate the MoCap technology for gait analysis in a hospital rehabilitation service. The methodological approach was based on contrasted methodologies from the field of service design, which were materialised through observation techniques (during system use), semi-structured interviews, and workshops with healthcare professionals. A sample of 29 participants was analysed (13 patients, 10 ‘proxies’, and 6 doctors). The reporting of the results follows the Consolidated Criteria for Reporting Qualitative Research (COREQ) statement [37] (S1 Table).

### Paradigmatic position

Within the paradigms ‘that guide disciplined research’ [38], the paradigmatic position of this research is in the field of interpretivism (aligned with qualitative research). In interpretivism, ‘reality’ is constructed in people’s minds and can be clearly understood through an interactive dialogue between the researcher and participant [39]. The interpretivism paradigm and the qualitative methods are naturally closer than quantitative methods to design practice [RW.ERROR—Unable to find reference:453]. According to Blanco et al. [40], not considering the qualitative perspective of end users could lead to suboptimal solutions in product and service designs. In our case, the research question of this study is concerned with providing an understanding of how to integrate MoCap gait analysis technology into the rehabilitation field according to the end user’s knowledge, experience, and expectations.

### Ethics

The experimental study was conducted after the formal approval of the local ethical committee (Bioethics Committee of Aragón Spain, CEICA; Act No. 12/2018). The study was conducted in accordance with relevant ethical guidelines, including a verbal explanation and written informed consent from the participants.

### The gait analysis system

The MoCap system used in this study was the Move-Human Sensors system developed by the IDERGO research group, which includes a module for gait cycle analysis [15,41]. The first version of the system was implemented in 2007. Since then, it has been used in numerous public and private projects both in hospitals (healthcare field) and companies (ergonomics field). The system has been incorporating the concerns of the professionals involved in the projects (engineers, doctors, ergonomists, prevention technicians, etc.).

The measurement validity of the MoCap system is largely determined by the accuracy of the sensors it uses. In this case, it is based on wireless IMUs, specifically, the NGIMUs devices [42], which are placed on the patient’s body with elastic bands. The NGIMU sensors are calibrated by the manufacturer. They filter and process the signal internally to directly send the rotation information. The NGIMU sensors have been used and assessed in numerous publications, which have guaranteed their accuracy, as can be found on the manufacturer’s website [43].

The gait test of this study is aimed to be used as a medical test based on pre- and post-measurement sessions for the applied rehabilitation treatments. The generated reports show the changes that have occurred in each patient’s gait between the pre- and post-sessions. These reports can be used to make decisions about the treatments (continue treatment, change to another, increase the intensity, etc.). In this regard, it should be noted that this study does not focus on the gait report, how it is designed, or what information it contains. Instead, we present a more global perspective of the service design. However, because the gait report design is a relevant research issue, we have considered it in the fieldwork to extract participants’ expectations and research opportunities.

### Hospital setting

The research setting was the Rehabilitation and Physical Medicine Service of the Miguel Servet University Hospital (HUMS) of Zaragoza, Spain, a reference hospital in the country. This service provides rehabilitative assistance to return the highest degree of functional capacity and independence to the patient as possible, favouring family, social, and work reinsertion. It is organised into areas of hospitalisation, outpatient consultation, and therapy (physiotherapy, occupational therapy, hydrotherapy, electrotherapy, and speech therapy).

### Pathology and treatment

We were focused on evaluating the gait of patients with spasticity when they receive treatment with botulinum toxin. The choice of these patients makes it possible to extrapolate the results to other patients with more favourable physical or cognitive conditions. Spasticity is a symptom that affects a large group of patients after suffering from neurological damage. Negative effects include pain, decreased mobility, contractures, and muscle spasms, which can interfere with daily life activities and sleep to a greater or lesser degree. The causes are diverse; some of the best-known causes are stroke [44], multiple sclerosis [45], post-traumatic brain damage [44], spinal cord injury [46], cerebral palsy, amyotrophic lateral sclerosis, and polyradiculitis. In HUMS, more than 850 patients were admitted with stroke pathology in 2017, and 75% of these cases required attention and subsequent follow-up.

The botulinum toxin treatment aims to treat focal spasticity via muscle infiltration with reversible paralytic action after 4 to 6 months [44,47,48]. Although there are other possible treatments for spasticity, for this study, the observed efficacy, personalised patient needs (dose, muscles to be inoculated, etc.), relatively high cost, and widespread use of the treatment justify the treatment choice. The gait test can aid the doctor in decisions [49] regarding (1) continuing to apply the treatment, (2) detecting whether the infiltrated muscles are adequate, and (3) maintaining or modifying the dose.

### Study design

Discover, define, design, and develop are the most common phases of service model development [28], constituting the global structure and philosophy that we follow. However, in this paper, as claimed in the literature [33], we delved into the specific methods related to our case study, providing the methodology design, reasoning, and how the specific methods were used and applied. Consistently, we contextualised the study design through a theoretical framework based on methods endorsed by the scientific community.

**Fig 1:**
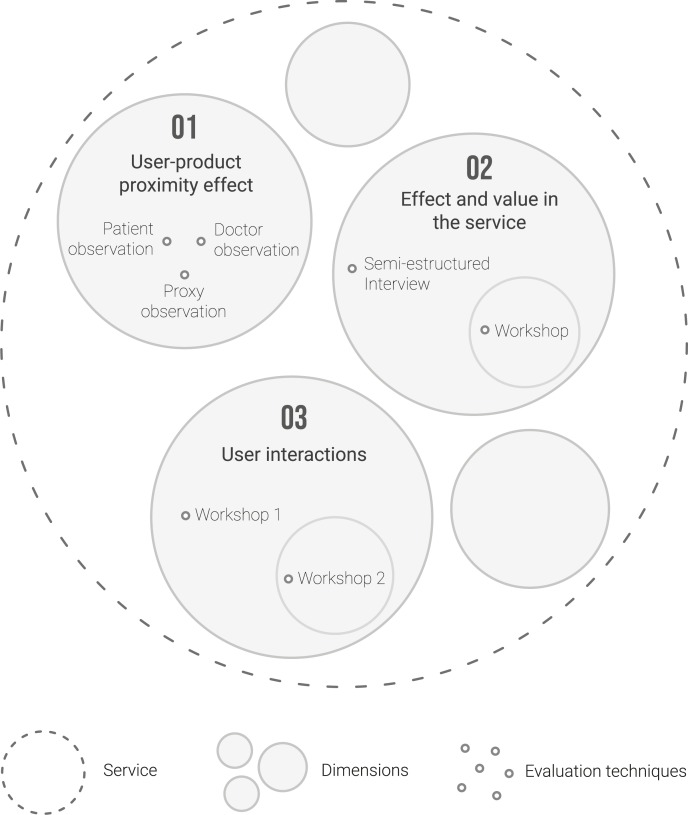
Product evaluation strategies in our case study, based on Blanco et al. [40].

Our scenario started from an existing gait test based on IMU technology. In this context, we proposed a methodological approach to qualitatively assess three dimensions of the test, which gave rise to our research phases: (1) the user-product proximity effect, (2) the effect on and value in the service, and (3) user interactions. The theoretical framework of this approach was based on the following contrasted methodologies from the field of service design and HCD (which, as we have seen, is related to the PCC philosophy):

- The general vision was based on the Octopus methodology by Marin et al. [15], which aims to define design specifications to create MoCap devices from three points of view: product, software (information analysis), and service. As the design object is a service, this study focused on this approach.
- The phases within the methodological sequence were based on the Xassess evaluation methodology by Blanco et al. [40]. Because the starting point was an existing product (a gait analysis system), we inevitably faced the evaluation of the system. In this regard, Xassess proposed three evaluation strategies: (1) ‘complementation’ (each product dimension is evaluated with one qualitative or quantitative technique), (2) ‘triangulation’ (each dimension is evaluated with two or more parallel techniques), and (3) ‘combination’ (each dimension is evaluated with two or more successive techniques). As shown in Fig 1, the methodological proposal followed a general strategy of triangulation (three parallel phases). Examining each phase separately, Phase 1 followed a triangulation strategy, and Phases 2 and 3 followed a combination strategy. Combination strategies offer advantages because each evaluation illuminates the following steps or techniques strategies, avoiding overlap and favouring the construction of knowledge on a solid basis.
- Finally, Phase 3 was based on Community, which Blanco [26] proposed as a methodology for the design of complex services with interrelationships between multiple users. Here, it was adapted to a workshop format for professionals from the health sector.

Table 1 shows the construction of the research methodology. Three assessment dimensions (phases) led to concrete research questions, which were answered with different user profiles (participants) through observation techniques, semi-structured interviews, and workshops.

**Table 1:** Research methodology.

	Dimensions to evaluate	Research questions	Research techniques	Participants	Main output results section
**Phase 1**	User-product proximity effect	How do the actors involved react to the test? How can we consider their experience for the gait test design?	Gait test Observation: 26 sessions	13 patients, 10 proxies, 6 doctors of the service (head of the service, head of the neurological section, 3 specialists, and 1 resident)	First 5 subsections
**Phase 2**	Effect and value in the service	How can the test be integrated into the service? What route will patients follow in the service?	Semi-structured interview: 1 session Workshop: 1 session	2 doctors of the service (head of the service and head of the neurological section)	Diagram 1 of the 6th section
**Phase 3**	User interactions	Which actors are involved directly or indirectly with the test? What information flows exist or should exist between them?	Workshop: 2 sessions	The 6 doctors from Phase 1	Diagram 2 of the 6th section

Regarding the participants mentioned in Table 1, the relationship established with the doctors was possibly due to previous meetings in which they showed a shared interest in integrating a gait analysis system into their service. A relationship was established with the patients with the six doctors in Table 1, who conducted the patient rehabilitation and recruited them face-to-face during the consultation. The interest in improving rehabilitation through gait analysis was communicated to the patients and proxies. No one refused to participate in the study.

The sample size of patients and proxies (phase 1) was determined by the concept of *saturation*, which was defined by Glaser and Strauss [50] and has been widely used in qualitative research. Saturation has been reached when adding more participants to the study does not generate additional insight or information. In this way, the measurement sessions were repeated until saturation was reached with 13 patients and 10 proxies. The sample size of doctors was six (Phases 1, 2, and 3), which corresponds to the number of physicians involved in the analysed rehabilitation service and the applied treatment.

In the following sections, each phase of the methodology is explained in depth, including the specific research objectives, participants, and study design.

### Phase 1: User-product proximity effect

Phase 1 aims to learn from observing the use of the MoCap system in its context. The strategy in this step was to perform the gait test in the rehabilitation service, carrying out an observation focused on understanding the effect on the involved actors (patients, proxies, and professionals).

There were 26 observation sessions carried out with 29 users of different profiles: the patients (*n* = 13) who performed the test, the proxies (*n* = 10) who (in some cases) accompanied the patients and observed the test from nearby, and the doctors of the service (*n* = 5 rehabilitation specialist doctors and 1 resident doctor) who were free to observe the gait test and talk with the patients or proxies. Each of the 13 patients with spasticity (7 men and 6 women; average age = 45.9 ± 19.8 years) underwent the gait test twice. The first test was performed a few minutes before receiving the botulinum toxin treatment, and a follow-up was performed a month later.

The patients had been diagnosed by the rehabilitation service of the hospital in the evaluation consultation and were selected for the study by meeting the following inclusion criteria:

- The patient can walk autonomously.
- The patient presents a dynamic or reducible contracture that alters motor function.
- A reduction in spasticity is expected to lead to a functional improvement with the treatment, according to the doctor’s previous experience.
- The patient has the possibility of receiving periodic controls to learn patterns of movement at home or complementary physiotherapy treatment.
- The patient is included in the indications of the technical sheet approved by the Spanish Agency of Medicines.

Table 2 includes the patient’s characteristics. The gait speed at the beginning of the patient’s evaluation has been included because it is an important indicator to represent the general state of health and is related to impairment, functionality, mobility, independence, autonomy, and comorbidity levels [51,52].

**Table 2:** Patient’s characteristics.

ID	Affected side	Gender	Age	Height [cm]	Abdominal Perimeter [cm]	Initial Gait speed [m/s]	Proxy
P001	L	M	36	177	108	0.36	Yes
P002	R	M	19	170	85	0.86	Yes
P003	R	M	44	172	85	0.79	Yes
P004	R	F	55	161	115	0.30	Yes
P005	L	M	18	164	64	0.66	Yes
P006	R	F	32	164	90	0.77	Yes
P007	L	F	69	148	83	0.19	Yes
P008	L	F	63	154	106	0.37	Yes
P009	L	M	70	176	102	0.34	Yes
P010	L	M	19	173	96	0.68	Yes
P011	R	M	60	164	92	0.33	No
P012	R	F	44	170	98	0.34	No
P013	R	F	68	165	92	0.32	No
13 patients	7 (R), 6 (L)	6 (F), 7 (M)	45.9 (19.8)	166 (8.4)	93.5 (13.1)	0.49 (0.23)	10 proxies

According to the statement terminology of the COREQ [37], the sampling method was purposive in the case of the patients because they met the inclusion criteria and were of diverse ages and disease levels, according to the doctor’s criteria. Proxies were recruited through the snowball method because they were dependent on the patient section. Doctors were also purposively sampled because they represented different profiles (two heads, three specialists, and a resident doctor).

**Fig 2:**
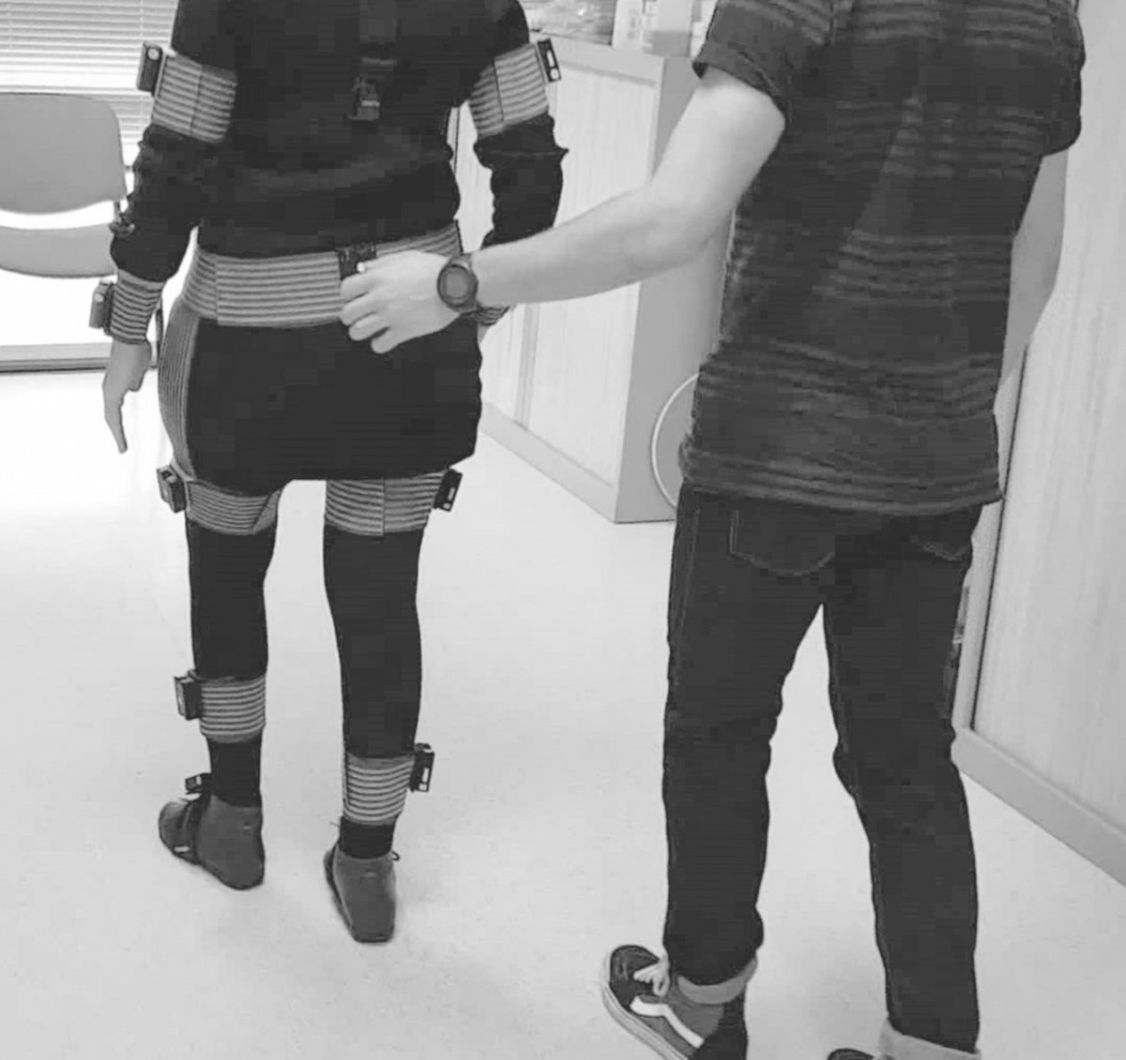
Gait test in hospital. The individual in this picture has given written informed consent (as outlined in the *PloS* consent form) to publish these case details.

The gait test was carried out using two ‘test guides’, one engineer (JM), who managed the computer, and a physiotherapist (AM), who guided the patients and interacted with them. In each measurement session, the patients were instrumented with the MoCap sensors (Fig 2) and walked naturally 6 m in a straight line at a self-selected speed. When the distance was completed, the patient turned around and walked back in the opposite direction. This operation was repeated to measure up to 25 strides. Only strides in a straight line were used, discarding any turns and start and stop zones. The duration of each test was 20 to 25 minutes.

The observations during the test were carried out by the test guides. It was established that observers should focus on the following factors or dimensions: (1) physical and cognitive abilities (patients), (2) motivation (patients), (3) concentration (patients), (4) reactions to the test and technology including what they said, what they did, and how they responded (patients, relatives, and doctors), and (5) operational and technical problems (test guides). Field notes were made during the observation. Audio and visual recordings were not used because the pre-defined observation dimensions were considered enough to address the research objectives.

### Phase 2: Effect and value in the service

Designing a system that aims to advise professionals in the rehabilitation process requires going beyond what happens only in the test session. Phase 2 evaluates the effect and value of the micro-service within the HUMS macro-service, seeking to obtain an overview of the general path followed by the patient according to the diagnostic and care decisions.

Regarding the participants involved in this phase, owing to their deep understanding of service performance, the participants were two doctors who were familiar with the gait test: (1) the head of the neurological rehabilitation section and (2) the head of the rehabilitation service.

First, a face-to-face semi-structured interview with the user (1) was conducted, addressing the issues collected in Table 3. This allowed us to develop the first version of a graphic map that represents the flow of clinical decisions of the service with the new test introduced in it.

**Table 3:** Semi-structured interview questions.

How is the service structured?
What steps do patients follow when they receive the service?
What are the logistics, periodicity of the reviews, and communication pathways between patients and healthcare professionals?
What decisions do physicians have to make regarding patients?
What is the parallelism of the test with other existing medical tests?
Which healthcare professional should perform the test?

Following a ‘combination’ strategy [40], in the second session, a workshop was held with both users (1 and 2) to evaluate the map of the service proposed after the first interview. The users had the opportunity to redefine and improve it to ensure that it reflected the entire process. The duration of each session was 40 minutes. They were pilot tested and conducted by JM and AM who recorded the audio.

### Phase 3: User interactions

We assume that we are facing a complex and multi-user information network. For this reason, Phase 3 aims to define the information flows (connections) that must exist between the macro-service users for an adequate implementation of the gait test. These connections should allow the test implementation and should cover the relational needs of the Community methodology [17], improving the user experience, acceptance, and clinical effectiveness of the new gait test.

A workshop was held twice in succession with six doctors from the rehabilitation service, as shown in Table 1, first with three doctors and later with another three others. They were pilot tested and conducted by JM and AM. The duration of each workshop was 100 minutes, and the audio was recorded.

**Fig 3:**
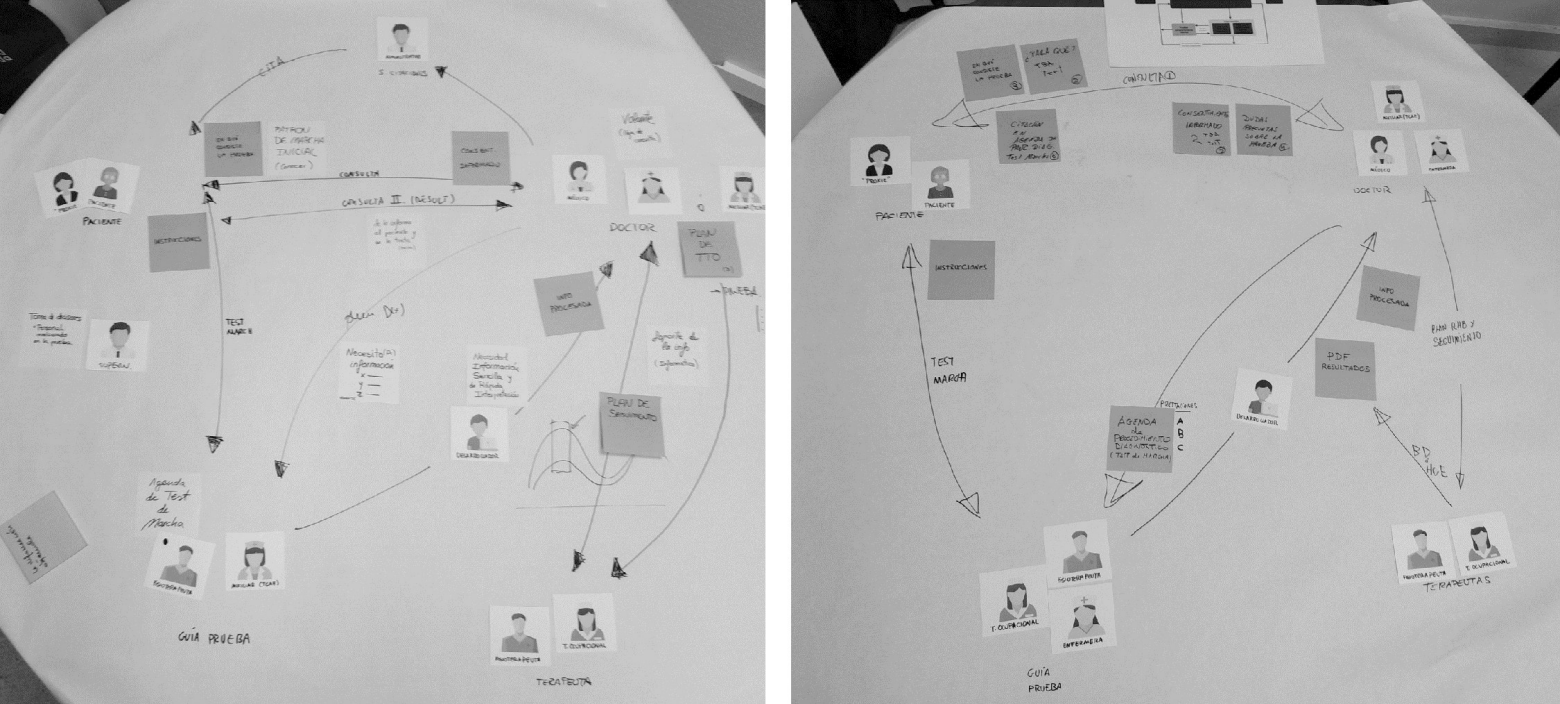
The maps of relational needs from the workshops with the doctors.

The workshop challenge was ‘How could we develop a useful gait test for the rehabilitation service?’. The main task involved collaboratively drawing the users’ relational needs by connecting user groups using arrows (unidirectional or bidirectional) that represent information exchanges and/or interactions. To facilitate the process, pre-designed cards with icons of the involved professionals had been prepared. Initially, the organisers structured some user groups and connections as an example. The schemes created by the doctors during the sessions are shown in Fig 3. In the second workshop, to reach a consensus between both workshops, following a ‘combination’ strategy [40], the researchers inquired about the differences from the map developed in the first session.

### Data analysis

Observational notes of Phase 1 and the audios of Phases 2 and 3 were transcribed and coded using the thematic analysis approach [53]. The full transcription was read several times separately by JM, JJM, and TB to identify differences and similarities of the content. Similar content was underlined in the same colour, and a descriptive concept (category) was assigned to each colour. Afterwards, the researchers discussed their reflections. Once a consensus was reached, the latent content and implicit messages of each category were described in the results section. According to the COREQ statement [37], the identified categories were derived from the data (i.e. they are inductive). To improve the understanding of the maps from Phases 2 and 3, they were laid out as simply and perceptibly as possible. It was necessary to hear the audios repeatedly to include all the comments in the maps, not just the handwritten information. Microsoft Word and Excel were used to manage the data, and Illustrator was used to create the figures.

### Trustworthiness

To achieve scientific rigour in qualitative research, Guba and Lincoln [38] proposed including a section about the trustworthiness of the interpretations. An integral aspect of trustworthiness is maintaining a detailed audit trail. Thus, we recorded our reflective memos and analysis decisions throughout the study. Additionally, in this paper, we provide clear and thick descriptions of the context, data collection, and analysis process, which favour the reproducibility of the study. To consider different perspectives and avoid bias, members of the research team represented different professions, and three of them independently identified and agreed on the categories presented in the results section. Finally, the resulting maps from Phases 2 and 3 reduced the subjective nature of the paper because they are tangible and factual objects proposed by the participants, who constitute an additional perspective as experts on their own experience in the environment [31].

## Results

From the field research, design considerations and guidelines have been obtained and grouped in the following sections: (1) patients’ understanding, (2) guiding the gait test, (3) which professionals guide the gait tests, (4) gait test reports, (5) requesting gait tests (doctors and test guide communication), and the (6) conceptual design of the service with the gait test.

### Patients’ understanding

The extreme diversity of the patients is evidenced. One of the most repeated phrases among doctors is ‘each patient is a whole world’ on a physical and cognitive level and in terms of the care that each one requires.

- **The accessibility level of the gait test should be maximised**.

Most patients receive other types of therapy than just those received in the hospital, such as therapies in elderly centres, associations, or private centres: ‘I've been going to rehabilitation since I can remember’. They are aware of their limitations and are realistic about their situation: ‘You can notice evolution for a while, but then you stabilise at a certain level’. ‘In winter, I can feel my muscles [are] more contracted’. However, we also observe how doctors try to lower the expectations that the proxies sometimes have with botulinum toxin therapy. They indicated that rehabilitation objectives that are too high or optimistic can lead to frustration and disinterest in the rehabilitation process.

- **It should be assumed that not all patients will be able to recover their previous functional capacity. They usually know their own limitations. We should take this into account and be honest in relation to their chances of recovery**.

Those who have decided to participate in this study go to rehabilitation voluntarily and have a clear motivation to recover; however, the degree and origin of this motivation are very diverse. Some of them transmit their willpower with their daily habits, ‘every morning I walk around the block’. ‘I am always trying to improve, stretching and moving, for example, while I cook or while I dress’. Those who are older show that they have recovered the motivation they had lost: ‘Recently, we went to the neurologist, and he explained the treatment to us. We have been encouraged to try. We had been disconnected from this world for many years’. In the youngest patients, motivation, strength, and interest usually come from family members, who are the protagonists in the interactions with the test guides and express their curiosity regarding new treatments and techniques.

- **The motivation of the patients and proxies should be exploited and maximised as much as possible. The gait test should become a motivating and engaging element whether the results are positive or negative**.

### Guiding the gait test

**Fig 4:**
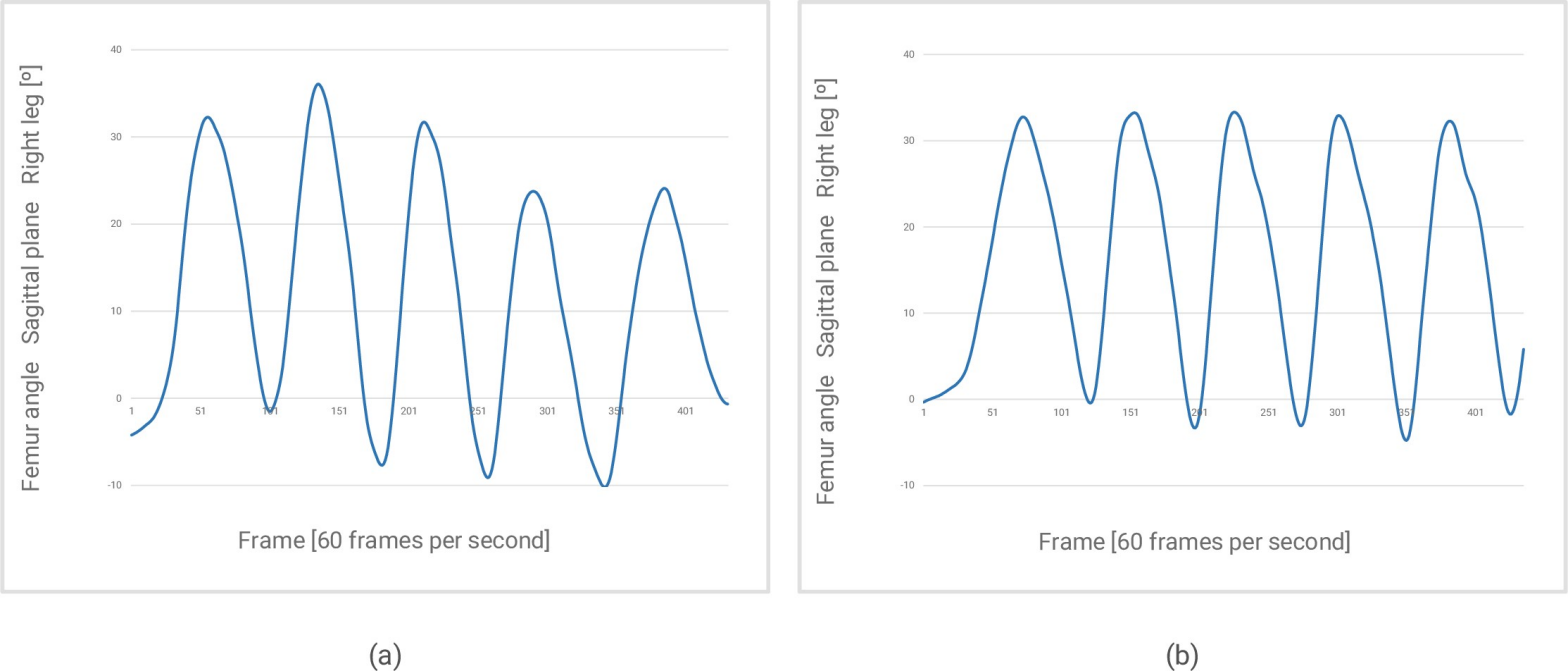
Femur angle with respect to the hip in the sagittal plane. (a) Before and (b) after the patient adapted to walking using the usual pattern.

How and who performs the test can be keys to its success. The test guides should ensure that patients walk with their usual pattern. In Fig 4, we see the movement curve irregularities of (a) a patient who is not accustomed to walking in the test and (b) those of the same patient who became accustomed to walking in the test after crossing the corridor two or three times.

As a general rule, the concentration level of the patients during the test was high. They were previously silent, struggling, and looking at the ground as they walked. Even those with limited cognitive and communicative ability understood the explanations and satisfactorily executed the test guide instructions. However, there are factors that affect the concentration and are necessary to consider to accelerate the tests and avoid incidents that would require discarding the results.

The patient’s concentration is improved with a calm and constant environment without sudden noises, foot traffic, or conversations. Patients can sometimes lose concentration, become scared, and even stop walking when someone suddenly enters the room, when there is a slam, or when staff or family members converse, more so if the conversation deals with issues related to the patient. To create this environment, the test guides must empathise with the patient and their relatives, using appropriate language with clear, concrete, and predictable instructions, while avoiding technical terminology (e.g. using anatomical calibration functions: ‘Now I need you to be very still, like in a picture’).

- **The role of the test guides is especially relevant. They should be able to extract the usual walking pattern of the patients. Access should be restricted to the room to achieve a calm and favourable environment during the performance of the test**.

During the tests, the patients displayed different emotions and reactions from showing fear of pain to satisfaction or gratitude. Considering these reactions can provide value from the user-experience point of view. Below, the reactions are sorted in the order of appearance along the process.

- **Mistrust, does it hurt?** One of the first reactions and doubts that arose in both patients and family members was regarding whether the test was painful. They worried about whether the devices give electric shocks or punctures. Many of them had suffered from various painful treatments, and these doubts could be a reason for the initial rejection.
- **Arriving late, anxiety**. Some were nervous about being late for the tests, either due to not finding the room, transportation difficulties, or the schedules of the relatives who accompanied them. The doctors indicated that sometimes patients present anxiety for these reasons, and they must spend the first minutes of the consultation session reassuring them. Nonetheless, this was not the case in this study.
- **Fatigue**. Some patients, owing to their physical condition and especially their age, asked to rest as soon as they reached the room. In these cases, the sensors were placed and removed while they were sitting.
- **Feeling observed**. Once we placed the sensors, we saw how the patients joked with their relatives. Others, especially the younger ones, presented a certain shyness and appeared to feel uncomfortable, exposed, and observed.
- **Gratitude**. At the end of the test, gratitude reactions often arose: ‘It is a very difficult disease. It is comforting to see how people are working on this. We will help with everything that we can’. It is important that they feel they are part of the technological evolution in this field.
- **Bond of trust in the second session**. As a general rule, on the second day, the patients were more confident. They interacted more and had a greater link with the test guides. The most noticeable effect occurred in the young patients, who, as mentioned, were uncomfortable and shy on the first day.
- **The test guides should explain the test properly and act effectively towards the different reactions that patients may present. Behaviour guidelines for test guides should be established**.

### Which professionals should guide the gait tests?

Due to the test characteristics, doctors concluded that two people are needed to run the test: one to guide the patient (place and remove sensors and give instructions to the patient) and another to operate the computer. Different health professionals have adequate training and capacity (doctors, nurses, physiotherapists, occupational therapists, etc.). According to healthcare professionals, in our environment, the test would be carried out by the nursing section: ‘Nursing is accustomed to doing this type of test; it falls within their competence’. ‘Certainly, there are tests performed by a doctor, for example, an endoscopy, but in that case, you are getting inside a human being. This test has nothing to do with that’.

Likewise, tests could also be performed by physiotherapists. These professionals are the most interested (along with doctors) in obtaining objective information on the gait pattern because they also apply therapies aimed at rehabilitating walking; however, the availability of these professionals is reduced, at least at HUMS. The final decision rests on the service head, on the professional availability of each hospital, and on the derived costs.

- **The test should be conducted by two trained healthcare professionals. It would be feasible for nurses or physiotherapists to conduct the test. The decision depends on each service**.

### Gait test reports

Doctors have a negative perception of the results provided by the test. They consider the test to be far from their area of knowledge and difficult to interpret, requiring a high learning curve. They called it ‘the test of the engineers’, in some cases, showing concern and anxiety regarding who would interpret the data and how much time would be necessary to do so.

- **The gait analysis test should provide a brief and easily interpreted report. Results must be communicated to patients in an understandable and personalised way to make more consensual decisions**.

Defining the content and design of the test report is one of the challenges that must be addressed. According to the doctors’ opinion, the first test should serve to assess the functional state of the patient and, together with the rest of the information and inputs (clinical exploration, interview with the patient, clinical history, etc.), establish the rehabilitation objectives. Regarding the second test, they affirm that it should measure the treatment effects and allow the assessment of the achievement of the initial objectives: ‘We could try to assess if the patient has improved above what is expected, at what is expected, or below what is expected’.

- **Future research should serve to detect the most representative and useful information for clinical decision making**.
- **With the first test, the rehabilitation objectives are established. With the second test, whether the treatment effects were as expected is assessed**.

A general statement was made that the test report results should be available in digital format for consultation between the professionals involved in a patient’s rehabilitation. Consequently, the report should be uploaded and incorporated into the patient’s electronic medical record. In this regard, HUMS has its own intranet and another at the regional level. In our research, we refer to the electronic medical record without differentiating between these two. In this sense, the communication vehicle between doctors and therapists is the electronic rehabilitation plan, which allows prescribing treatments and tracking the patient by the involved physicians (renamed the electronic monitoring plan once the treatment begins). Uploading the gait test report to the electronic medical record would improve the doctor-therapist communication so they could share considerations about the results through the electronic rehabilitation plan and take more consensual decisions based on objective information.

- **The doctor should be able to upload the gait test report in digital format to the electronic medical record**.

### Requesting gait tests: Doctors and test guide communication

Doctors agreed that it is not possible to run the test during consultation time. According to them, ‘it is the same as an X-ray or a blood test; I prescribe it, and I get the results back’. One of the questions that emerged in the workshops was how a doctor could request a test from the consultation. In this regard, doctors proposed the creation of a new agenda called the ‘gait test agenda’ with specific schedules and professionals assigned that would be shared between doctors and test guides and completed in the consultation.

- **To cite prescribe patients to perform the test, it is proposed to create an electronic ‘gait test agenda’ shared between doctors and test guides**.

### Conceptual design of the service with the gait test

**Fig 5:**
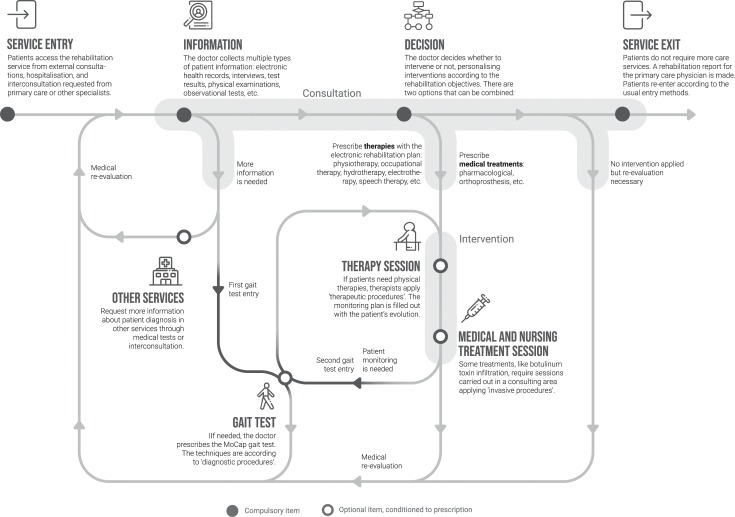
Actuation flowchart decision making of the rehabilitation service. Figure elaborated by authors. Icons made by Freepick, Pause08, Pixel perfect and Srip from www.flaticon.com.

Based on the specifications obtained in the previous sections and according to the specific outputs of Phases 2 and 3 of the methodology (Table 1), two schemes are presented that represent a conceptual proposal of the rehabilitation service that includes the new gait test in its operation. The first scheme, called the actuation flowchart (Fig 5), shows the circuit of decisions that physicians must face during the spasticity rehabilitation process. The figure shows two different areas shaded in grey: consultation and intervention. A patient will go through both areas iteratively as many times as necessary. It also shows the gait test, which is accessed through two entryways:

- **First gait entry**. The test can be prescribed from a consultation in which the doctor considers that further information is needed. In this case, the test would have a diagnostic purpose. This option is parallel to the current possibility of requesting information from other services (e.g. an interconsultation request to obtain a psychological report).
- **Second gait entry**. It is possible to incorporate the gait tests during the intervention process, as long as it is planned from the consultation. In this case, the test would have a monitoring function. This option, based on pre- and post-treatment measurement sessions, is the one planned (methods section) to assist clinicians during the rehabilitation process.

Note that an item drawn with an empty circle indicates that the patient will undergo the process only if it is prescribed by the responsible physician (e.g. a patient may receive therapy sessions but not receive medical and nursing treatment sessions).

**Fig 6:**
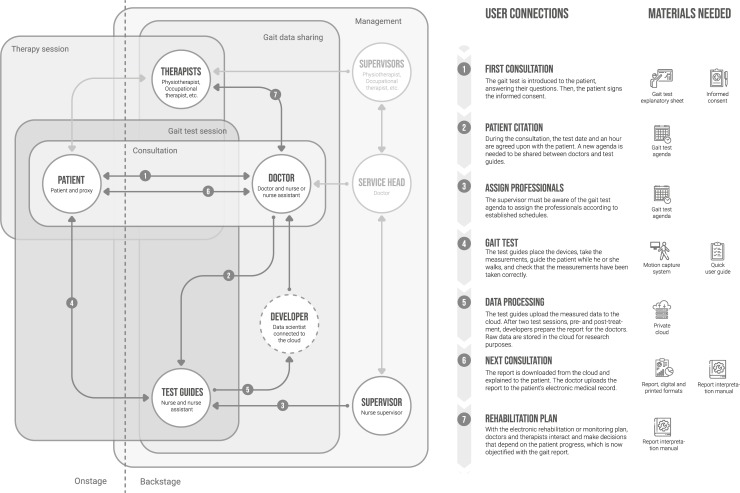
Community scheme based on [26] with new or improved information flows to include the gait test in the service. Figure elaborated by authors. Icons made by Freepik, Smartline, Mynamepong, Pause08 and dDara from www.flaticon.com.

The second scheme, developed following Community [26], represents the connections between the user profiles that interact with the gait test and its results (Fig 6; arrows from 1 to 7). Dark arrows represent the information flows that need to be designed and implemented. Light coloured lines represent the macro-service connections, which our design will not influence, but it will have a certain influence on the gait test integration. The represented users are grouped according to the ‘network spaces’, areas of conceptual interaction with different objectives and themes where users communicate and interactions can take place (e.g. gait test session, management, etc.). We see how a user can be present in several ‘network spaces’.

Likewise, in the scheme, the materials to be developed are established (graphic documents, electronic agenda, cloud, etc.), which are also called ‘touch points’, that is, tangible or intangible elements that are in contact with the users. In line with the theory of service design, two conceptual interaction areas are also included, ‘backstage’ and ‘onstage’ [54]. Backstage is the invisible part for the user, where the processes that articulate the services take place. Onstage is the visible part that encompasses the target users.

Note that ‘developers’ have been included as temporary participants in the process because, although their permanent presence could be useful, their role during the first stages will be to process the information manually. Once the project evolves, and it is decided which information is the most adequate to facilitate the clinical evaluation, this process can be automated, and these users can be eliminated.

## Discussion

This study highlights an underlying need for MoCap gait analysis technology. Although it can generate individual objective outcome measures in usual clinical practice [55], further research is required for its effective and useful clinical application. In this framework, we apply a research methodology that starts from a specific concept: to design a gait test integrated into the hospital setting that provides objective information to support decision making regarding the rehabilitation process. We focus on the case study of neurological patients with disorders derived from spasticity who receive treatment with botulinum toxin. We have conducted a field investigation introducing a gait analysis system into the HUMS environment and evaluated it through observation, interviews, and workshops. As a result, design considerations have been obtained that can be useful for design teams that face these types of challenges in the future. Among these considerations, the micro-service (gait test) integrated into the macro-service (hospital rehabilitation service) has been conceptually defined through two infographics.

We assume that the proposed concept involves an extra effort for hospitals (economic and resource investment), for professionals (more workload and learning), and for patients and proxies (more visits to the hospital). However, the gait test would provide a substantial improvement in the quality of care, encouraging professional development and collaborative research among physicians. Likewise, the system requirements are relatively simple. With a short test duration and a short preparation time (20–25 min per patient), it does not require an exclusively dedicated room. For patients, it is not an invasive test, and it does not entail visiting centres other than the hospital where they usually receive treatments. The advantages and benefits that it would bring in rehabilitation services are described below:

- **Sustain decisions at a clinical level**. Having a test that provides objective data on the patient therapy response allows more precision in the decision-making process, including the option of not further intervening.
- **Sustain decisions at the administrative and social levels**. Inevitably, physicians are under pressure that comes with human responsibility, schedules, efficiency requirements, legal disputes, or limitations in resources.
- **Improve treatment profitability**. Some treatments entail significant costs to the public or private health entities involved, as in the case of treatment with botulinum toxin. The gait test can avoid applying those treatments that do not provide positive effects to certain patients.
- **Facilitate communication between physicians**. The test could unify gait evaluation and enhance the transmission of knowledge among health professionals.
- **Positive feedback for the therapist**. Objective information allows the clinical plan to be personalised for each patient, establishing more realistic objectives and generating a theoretical basis that could improve their evolution.
- **Positive feedback for the patient**. It may involve extra motivation for patients, encouraging confidence in the treatments and a greater perception of healthcare safety and trust.
- **Improve medical and patient communication**. Properly presented information can establish a doctor-patient communication pathway, improving the reasoning of patients (and relatives) and improving treatment decision making.
- **Feed databases**. Collecting gait information may have a practical effect to create predictions for new patients and facilitate diagnoses through machine-learning techniques.
- **Enhance research among physicians**. It can improve the collaborative research work among physicians, allowing the validation of new or modified treatments. Data from the database can be filtered for each investigation.

The mentioned benefits coincide with some concepts that McHorney and Tarlov [56] generically described for the healthcare field sector. They provide ways in which data from individual people in healthcare can be used, including to describe a patient’s health state, to monitor disease progression, or to standardize interactions between health care practitioners and patients [56]. In this regard, Cella et al. [57] summarised the concepts from McHorney and Tarlov [56] into two possible perspectives or uses that have similarities with the two gait test entries that we propose in Fig 5. The first use (similar to the first gait entry) is clinical decision making under uncertainty: ‘estimating the likelihood that a patient will profit from a given intervention’. The second use (similar to the second gait entry) is clinical evaluation: ‘examining whether a given treatment makes a meaningful difference for an individual patient’.

Beyond the benefits that MoCap gait analysis would bring in rehabilitation services, this paper has two main strengths or contributions. First, it provides design considerations and guidelines to integrate a gait analysis test based on MoCap technology in the rehabilitation hospital setting, which is a novel contribution and can encourage the use of this technology in this field. In this regard, Martin et al. [58] encouraged researchers to realise studies that propose design recommendations based on HCD, especially in scenarios where users (in our case patients) are heterogeneous (as we see in patients with hemiplegia). This reasoning supports the presented results.

The second strength is proposing a reproducible methodological approach to integrate a ‘micro-service’ (gait test) within a ‘macro-service’ (rehabilitation service), which advances service design knowledge and may be applied to other case studies or other technologies. This methodological approach had great acceptance among physicians. They ensured that the sessions where they participated were useful, practical, and different from the usual way of working: ‘I see these types of sessions and workshops as essential. It is useful to clean up many useless connections and make them simpler. I like it [the map]; it’s clean and illustrative’.

The importance of properly designing the rehabilitation service is manifested in numerous investigations that seek to assess the patient’s experience (e.g. [59,60]). In this context, service design and the theoretical basis of service-dominant logic [32] have great importance. They allow one to address stakeholder’s needs and strengthen professional relationships. However, as Han et al. [28] indicated, it is necessary to consider the specific needs of the clinical environment when applying service design techniques. This is translated to our specific case study in the necessity of integrating a ‘micro-service’ (gait test) within a more complex ‘macro-service’ (rehabilitation service). The scenario of the micro-service integration has particularities that differentiate it from a mere application of service design methods, which concern the design of the service as a whole. In return, we focus our design on ‘one link in the chain’, a new gait test in a hospital rehabilitation service. According to Wetter et al. [33], this contributes to improving the theoretical framework of the service design because it is not actually clear how to act at a ‘micro-level’ approach to connect design methods with real situations and favour service innovations.

Additionally, certain weaknesses or limitations have been identified in this study. It should be noted that we focus on a relatively specific case (gait tests for patients with spasticity in a particular rehabilitation service) and on a limited sample of professionals who know the service and the treatment used. Thus, if one intends to integrate this or another similar system into another hospital, it should be considered that other centres may be different at the organisational level. Some concepts (mentioned throughout the results section), such as agendas, the involved personnel, the rehabilitation plan, or the patients under study, may be different; thus, certain sections may be adapted to the particular case. In this regard, it may be useful to rely on the research methodology presented, especially in Phases 2 and 3.

Another limitation concerns the (1) gait report (including processing backwards) and the (2) measurement validity of the MoCap system. Although they are not part of the objectives of this study, they are key questions for the system design to achieve successful integration in a hospital. Both topics have been extensively discussed in the literature, and we encourage further investigation. In this sense, this paper is focused on the service design, which complements and strengthens this area of research.

Additionally, it should be noted that this study is interpretivist and the research techniques used are qualitative. This intrinsically implies that the participants are those who experience, process, and label the ‘reality’ under investigation and transmit it to the researcher [39]. To do this, the participants rely on their individual experience, memories, and expectations [38]. This prevents the total objectivity of the study and complete neutrality.

In summary, although we focused on a relatively specific case (gait tests for patients with spasticity in a particular rehabilitation service), the proposed design recommendations are partially generalisable to other treatments or health centres. Additionally, the proposed methodology can be useful and reproducible to obtain design considerations to integrate a micro-service into a macro-service.

Nowadays, healthcare technology is developing more rapidly and efficiently and requires fewer resources. This research contributes to improving the application of this type of system and other Internet of things devices [16] that can be developed in the healthcare field of ‘smart health’ [61,62]. It is expected that future health devices (micro-services) could be more useful to hospital services (macro-services) by maintaining their use over time and improving their acceptance.

## Conclusions

We conducted a field investigation introducing a gait analysis system into a hospital rehabilitation environment and evaluated it through observation, interviews, and workshops. We focused on patients with spasticity who received treatment with botulinum toxin. The main conclusion is that integrating a gait test into hospital rehabilitation services is beneficial for physicians, patients, and proxies, but to make it really applicable and useful, some design specifications should be considered. The design specifications and the methods applied to obtain them can be useful for both technology developers and healthcare professionals who seek to improve the quality of healthcare services.

## Supporting information

S1 Table Reporting according to the COREQ criteria. (DOCX)
