# Supplementary material for: Integrating a gait analysis test in hospital rehabilitation: A service design approach
Source: PLoS One. 2019 Oct 30;14(10):e0224409. doi: 10.1371/journal.pone.0224409 (PMC6821402; doi:10.1371/journal.pone.0224409)
Supplement: S1 Table — (DOCX) [file pone.0224409.s001.docx]

**S1. COREQ 32-item checklist**

Our research included three types of participants (patients, proxies, and doctors) through three research phases (Phases 1, 2, and 3). Thus, certain items are separately specified in the table according to the participants or the phases.

| **No. Item** | **Guide questions/ description** | **Comments** |
| --- | --- | --- |
| **Domain 1: Research team and reflexivity** | | |
| ***Personal characteristics*** | | |
| 1. Interviewer/facilitator | Which author/s conducted the interview? | JM and AM. |
| 2. Credentials | What were the researcher’s credentials? *e.g., PhD, MD* | JM: 3^rd^ year PhD Student. Design Engineering  TB: Professor, and PhD. Design Engineering  JJM: Professor, and PhD. Design Engineering.  AM: 2^nd^ year PhD Student. Physiotherapist.  EM: Medical Doctor.  JCA: Medical Doctor, and PhD. |
| 3. Occupation | What was their occupation at the time of the study? | JM: University Researcher.  TB: University Researcher.  JJM: University Research Group Coordinator.  AM: University Researcher.  EM: Public Hospital Rehabilitation.  JCA: Head of Public Hospital Rehabilitation Service. |
| 4. Gender | Was the researcher male or female? | 2 female and 4 male. |
| 5. Experience and training | What experience or training did the researchers have? | JM, AM, EM: > 4 years of qualitative research.  TB, JJM, JCA: > 15 years of qualitative research. |
| ***Relationship with participants*** | | |
| 6. Relationship established | Was a relationship established  prior to study commencement? | Patients and proxies: Possibly by doctors who conduct the rehabilitation (EM and JCA), and four more rehabilitation specialists (NR, RJ, IS, and TZ) mentioned in the acknowledgement section.  Doctors: Possibly by previous meetings with them. |
| 7. Participant knowledge of the interviewer | What did the participants know about the researcher? *e.g., personal goals, reasons for doing the research* | Patients and proxies: Verbal and written (information sheet) outlines were given.  Doctors: Shared interest to integrate a gait analysis system in the service. |
| 8. Interviewer characteristics | What characteristics were reported about the interviewer/facilitator? *E.g.,* *bias,* *assumptions, reasons and interests* *in the research topic* | Experience in the gait analysis system. Interest about the information that it provides to improve rehabilitation decisions. |

| **Domain 2: study design** | | | | |
| --- | --- | --- | --- | --- |
| ***Theoretical framework*** | | | | |
| 9. Methodological orientation and Theory | | What methodological orientation was stated to underpin the study? *e.g. grounded theory, discourse* *analysis, ethnography,* *phenomenology, content analysis* | The paradigmatic position of this research is situated within interpretivism (aligned to qualitative research).  The methodological approach aims to evaluate the gait analysis product/service to integrate it into the hospital rehabilitation environment.  Contrasted methodologies from the field of service design and HCD were used.  Content analysis was used to systematically organise data into a structured format. | |
| ***Participant selection*** | | | | |
| 10. Sampling | | How were participants selected? *e.g. purposive, convenience,* *consecutive, snowball* | Patient: Purposive. They met the inclusion criteria and were of diverse ages and disease levels, according to the doctor’s criteria.  Proxies: Snowball. Dependence on patients.  Doctors: Purposive. They represented different profiles (2 heads, 3 specialists, and 1 resident doctor). | |
| 11. Method of approach | | How were participants approached? e*.g. face-to-face,* *telephone, mail, email* | Patients and proxies: Face-to-face recruitment during rehabilitation consultation.  Doctors: Previous face-to-face meetings. | |
| 12. Sample size | | How many participants were in the study? | Phase 1: 13 patients, 10 proxies, and 6 doctors of the service (head of the service, head of the neurological section, 3 specialists, 1 resident).  Phase 2: 2 doctors of the service (head of the rehabilitation service and the head of the neurological section).  Phase 3: The 6 doctors from Phase 1. | |
| 13. Non-participation | | How many people refused to participate or dropped out? Reasons? | No one refused to participate in the study. | |
| ***Setting*** | | | | |
| 14. Setting of data collection | | Where was the data collected? *e.g. home, clinic, workplace* | At the rehabilitation service. | |
| 15. Presence of non-participants | | Was anyone else present besides the participants and researchers? | No | |
| 16. Description of sample | | What are the important characteristics of the sample? *e.g.* *demographic data, date* | Patients: People with hemiplegic spasticity who received rehabilitation though botulinum toxin treatment.  Proxies: Family, friends, or caregivers who accompany the patients.  Doctors: Medical doctors of the rehabilitation service, future users of the gait analysis system. | |
| ***Data collection*** | | | | |
| 17. Interview guide | Were questions, prompts, guides provided by the authors? Was it pilot tested? | | | Phase 1: Observation dimensions provided. Not pilot tested.  Phase 2: Semi-structured interview questions and workshop instructions provided. Pilot tested.  Phase 3: Workshop instructions provided. Pilot tested. |
| 18. Repeat interviews | Were repeat interviews carried out? | | | Phase 1: Yes, each patient underwent the gait test twice.  Phase 2: No.  Phase 3: No. |
| 19. Audio/visual recording | Did the researchers use audio or visual recording to collect the data? | | | Phase 1: No, pre-defined observation dimensions were enough to address the research objectives.  Phase 2: Yes, audio,  Phase 3: Yes, audio, |
| 20. Field notes | Were field notes made during and/or after the interview or focus group? | | | Phase 1: Yes, during the observation.  Phase 2: No.  Phase 3: No. |
| 21. Duration | What was the duration of the interviews or focus group? | | | Phase 1: 20-25 minutes each.  Phase 2: 40 minutes each.  Phase 3: 100 minutes each. |
| 22 Data saturation | Was data saturation discussed? | | | Yes. Sample size of patients and proxies was defined by saturation. Sample size of professionals was defined by the number of individuals who know or were familiar with the rehabilitation service and the applied treatment. |
| 23. Transcripts returned | Were transcripts returned to parti­ci­pants for comment and/or correc­tion? | | | Phase 1: No.  Phase 2: Yes, the workshop (2nd session) was based on the results of the first semi-structured interview and returned to the same participant.  Phase 3: No. |
| **Domain 3: analysis and findings** | | | | |
| ***Data analysis*** | | | | |
| 24. Number of data coders | How many data coders coded the data? | | | Three (JM, TB, and JJM). |
| 25. Description of the coding tree | Did authors provide a description of the coding tree? | | | No. |
| 26. Derivation of themes | Were themes identified in advance or derived from the data? | | | Derived from the data, i.e., inductive. |
| 27. Software | What software, if applicable, was used to manage the data? | | | Ms Word, Ms Excel, and Illustrator. |
| 28. Participant checking | Did participants provide feedback on the findings? | | | No. |
| ***Reporting*** | | | | |
| 29. Quotations presented | Was participant quotations presen­ted to illustrate the themes/findings? Was each quotation identified? *e.g. participant number* | | | Yes, we identified in the text which quotations came from patients, proxies, or professionals. |
| 30. Data and findings consistent | Was there consistency between the data presented and the findings? | | | Yes. |
| 31. Clarity of major themes | Were major themes clearly presented in the findings? | | | Yes. |
| 32. Clarity of minor themes | Is there a description of diverse cases or discussion of minor themes? | | | Yes. |
